# A scoring system to predict the risk of organ/space surgical site infections after laparoscopic gastrectomy for gastric cancer based on a large-scale retrospective study

**DOI:** 10.1007/s00464-015-4594-y

**Published:** 2015-10-20

**Authors:** Ru-Hong Tu, Chang-Ming Huang, Jian-Xian Lin, Qi-Yue Chen, Chao-Hui Zheng, Ping Li, Jian-Wei Xie, Jia-Bin Wang, Jun Lu, Long-Long Cao, Mi Lin

**Affiliations:** Department of Gastric Surgery, Fujian Medical University Union Hospital, No.29 Xinquan Road, Fuzhou, 350001 Fujian Province China

**Keywords:** Stomach cancer, Laparoscopy, Gastrectomy, SSI, Scoring system

## Abstract

**Background:**

A scoring system allows risk stratification of morbidity might be helpful for selecting risk-adapted interventions to improve surgical safety. Few studies have been designed to develop scoring systems to predict SSIs after laparoscopic gastrectomy for gastric cancer.

**Methods:**

We analyzed the records of 2364 patients who underwent laparoscopic gastrectomy for gastric cancer. A logistic regression model was used to identify the determinant variables and develop a predictive score.

**Results:**

There were 2364 patients, of whom 131 (5.5 %) developed overall SSIs, 33 (1.4 %) developed incisional SSIs, and 98 (4.1 %) developed organ/space SSIs. No significant risk factor was associated with incisional SSIs. A multivariate analysis showed the following adverse risk factors for organ/space SSIs: BMI ≥ 25 kg/m^2^, intraoperative blood loss ≥75 ml, operation time ≥240 min, and perioperative transfusion. Each of these factors contributed 1 point to the risk score. The organ/space SSIs rates were 1.8, 3.9, 9.9, and 39.0 % for the low-, intermediate-, high-, and extremely high-risk categories, respectively (*p* < 0.001). The area under the receiver operating characteristic curve for the score of organ/space SSIs was 0.734. There were no statistically significant differences between the observed and predicted incidence rates for organ/space SSIs in the validation set.

**Conclusions:**

This validated and simple scoring system could accurately predict the risk of organ/space SSIs after laparoscopic gastrectomy for gastric cancer. The score might be helpful in the selection of risk-adapted interventions to decrease the incidence rates of organ/space SSIs.

Surgical site infections (SSIs) are one of the most common nosocomial infections, accounting for 14–16 % of nosocomial infections overall and 38 % of nosocomial infections among surgical patients [1]. SSIs can lead to prolonged hospitalization, increased morbidity and mortality, increased surgery-related costs, and decreased quality of life [1, 2]. The incidence rates of overall SSIs after open gastrectomy are 7.0–20.8 % [3–6]. With an increase in the number of laparoscopic surgeries performed in gastric cancer patients, SSIs after laparoscopic gastrectomy have decreased compared with open procedures [7], but they are still one of the most serious concerns for surgeons and surgical patients. Therefore, the identification of patients who are at a high risk of SSIs might allow for the selection of a risk-adapted laparoscopic procedure and intervening perioperative measures to reduce SSIs, improve surgical safety and patient quality of life, and achieve the goal of being minimally invasive. The objective of the present study was to identify the risk factors for SSIs after a laparoscopic radical gastrectomy for gastric cancer in 2364 patients treated in our center. We aimed to use these risk factors to develop a scoring system for predicting SSIs.

## Materials and methods

### Materials

This study was a retrospective analysis of a prospectively collected database of 2364 primary gastric cancer patients treated with a laparoscopic radical gastrectomy in the Department of Gastric Surgery of Fujian Medical University Union Hospital, Fuzhou, China, between May 2007 and Jun 2014. The inclusion criteria were as follows: a histologically confirmed adenocarcinoma of the stomach; no evidence of tumors invading the adjacent organs (pancreas, spleen, liver, and transverse colon), para-aortic lymph node enlargement, or distant metastasis demonstrated by abdominal computed tomography (CT) and/or abdominal ultrasound and posteroanterior chest radiographs; and a D1/D1 + α/D1 + β/D2 lymphadenectomy with curative R0 according to the pathological diagnosis after the operation. The exclusion criteria were as follows: intraoperative evidence of peritoneal dissemination, invasion into the adjacent organs, or a distant metastasis; conversion to an open laparotomy; and incomplete pathological data. All procedures were performed after obtaining written informed consent following explanation of the surgical and oncological risks. The patient demographics, underlying diseases, clinicopathology, surgery data, and data on the preoperative and postoperative monitoring were recorded in a clinical data system for gastric cancer surgery [8]. The staging was performed according to the 7th edition of the UICC TNM classification [9]. The type of surgical resection (i.e., a distal subtotal gastrectomy, proximal subtotal gastrectomy, or total gastrectomy) and extent of lymph node dissection were selected according to the Japanese gastric cancer treatment guidelines [10]. Data were randomly assigned into two subsets using the SPSS version 18.0 (SPSS, Chicago, IL, USA), which were split 70/30: one for model development and the other for validation testing.

### Variables and definitions

We defined SSIs according to the surgical patient component of the 1999 Centers for Disease Control and Prevention (CDC) National Nosocomial Infection Surveillance (NNIS) System manual; [1] this definition includes incisional (superficial, deep) and organ/space SSIs. Briefly, superficial incisional SSIs were diagnosed within 30 days of the operation, confined to the skin and subcutaneous tissue, and associated with at least one of the following: pus, microorganisms isolated from culture of fluid or tissue, or signs or symptoms of infection. Either the surgeon or an attending physician made the diagnosis of a superficial SSI. A deep incisional SSI was diagnosed when the wound infection had spread to the fascia and muscular layers, but not the peritoneal cavity or pelvis (the organ/space), and one of the following criteria was also present: pus originating from the deep part of the incision, spontaneous wound dehiscence, or a wound opened by the surgeon. The surgeon made the diagnosis of a deep infection. Organ/space infections involved any organ or space other than an incised layer of the abdominal wall, such as the peritoneal cavity or pelvis.

The potential variables for SSIs were extracted from the database, including antibiotic prophylaxis (1 g of cefazolin was given 30 < 30 min before the incision, and an additional dose was given every 3 h during surgery), gender, age, body mass index (BMI, BMI ≥ 25 is considered as overweight, according to the World Health Organization classification [11]), previous abdominal surgery, Charlson co-morbidity score, perioperative transfusion (transfusion threshold Hb < 8.0 g/dl; maintenance range 8.0–9.5 g/dl), tumor location, tumor diameter, T stage, N stage, TNM stage, operation time (recorded from the skin incision to skin closure), intraoperative blood loss (estimated according to the volume of blood absorbed by the gauze and suction pumped after subtracting the volume of the fluids used for irrigation), type of surgical resection, type of reconstruction, D1/D1 +/D2 lymphadenectomy, and numbers of resected LNs.

### Statistical analysis

The continuous data were reported as the mean ± SD, and the differences between the groups were analyzed using *t* tests. The categorical data are presented as the proportion percentage and were analyzed with the Chi-square test or Fisher’s exact test. The variables with *p* < 0.05 in the univariate analysis were subsequently included in a multivariate binary logistic regression model. The variables that remained significant in the multivariate analysis were used to construct a scoring system to classify the patients into groups according to their risk of SSIs. A goodness-of-fit test was conducted to assess how well the model could discriminate between patients with and without SSIs. Model calibration, the degree to which the observed outcomes were similar to the outcomes predicted by the model across patients, was examined by comparing the observed averages with the predicted averages within each of the subgroups arranged in increasing order of patient risk. *p* < 0.05 was considered to be statistically significant. The statistical analyses were performed with SPSS version 18.0 (SPSS, Chicago, IL, USA).

## Results

### Clinicopathological characteristics of the patients

The clinicopathological characteristics of the 2364 patients are listed in Table 1. There were 1775 males and 589 females with a mean age of 60.93 ± 10.84 years. The average body mass index (BMI) of the patients was 22.20 ± 3.08 kg/m^2^. A total gastrectomy was performed in 1264 patients (53.5 %), distal gastrectomy in 1045 patients (44.2 %), and proximal gastrectomy in 55 patients (2.3 %); a D1 lymphadenectomy or D1 + lymphadenectomy was performed in 450 patients (19.0 %) and 1 914 patients for D2 lymphadenectomy (81.0 %); combined resection of other organs was performed in 17 patients (nine splenectomy: six for parenchymal injuries, one for splenic hilar vascular injury, one for splenic infarction, one for hypersplenism; three combined cholecystectomy for gallstone; three combined partial transverse colectomy for injuries; and two combined partial jejunectomy for injuries). The average surgery time was 180.86 ± 51.49 min, blood loss was 73.50 ± 104.04 ml, and the number of dissected lymph nodes per patient was 33.38 ± 12.96. According to the UICC TNM Classification of Malignant Tumors, 7th Edition, 477 patients (20.2 %) were in stage Ia, 216 (9.1 %) were in stage Ib, 242 (10.2 %) were in stage IIa, 264 (11.2 %) were in stage IIb, 239 (10.1 %) were in stage IIIa, 374 (15.8 %) were in stage IIIb, and 552 (23.3 %) were in stage IIIc.

**Table 1:** Univariable analyses of possible risk factors for the development of SSIs

Variables	No. patients	Incisional SSIs		Organ/space SSIs		Overall SSIs	
	(*n* = 2364)	(*n* = 33)	*p*	(*n* = 98)	*p*	(*n* = 131)	*p*
Age (year)			0.810		0.344		0.481
<65	1529	22		59		81	
≥65	835	11		39		50	
Gender			0.753		0.077		0.168
Male	1775	24		81		105	
Female	589	9		17		26	
ASA			0.255		0.461		0.953
≤2	2276	33		93		126	
>2	88	0		5		5	
BMI (kg/m^2^)		0.390		0.000		0.000	
<25	2026	30		62		92	
≥25	338	3		36		39	
Previous abdominal surgery		0.809		0.858		0.954	
Yes	347	4		15		19	
None	2017	29		83		112	
Charlson score		0.756		0.002		0.023	
0	1652	22		59		84	
1	492	5		34		39	
≥2	220	3		5		8	
Perioperative transfusion		0.191		0.000		0.000	
Yes	319	7		34		41	
None	2045	26		64		90	
Tumor diameter (mm)			0.406		0.698		0.930
<50	1634	25		66		91	
≥50	730	8		32		40	
Tumor location			0.998		0.644		0.761
Upper	614	8		26		34	
Middle	424	7		16		23	
Lower	1034	14		40		54	
≥2 areas	292	4		16		20	
T stage			0.603		0.877		0.939
T1	572	10		21		31	
T2	286	3		11		14	
T3	687	9		29		38	
T4a	819	11		37		48	
N stage			0.133		0.227		0.674
N0	888	15		29		44	
N1	341	8		14		22	
N2	382	2		22		24	
N3	753	8		33		41	
TNM stage			0.559		0.787		0.766
IA	477	6		17		23	
IB	216	5		6		11	
IIA	242	2		8		10	
IIB	264	6		13		19	
IIIA	239	4		11		15	
IIIB	374	4		16		20	
IIIC	552	6		27		33	
Operative time (mins)		0.261		0.000		0.000	
<180	1625	27		46		73	
180–240	519	4		22		26	
≥240	220	2		30		32	
IBL (ml)		0.914		0.000		0.000	
<75	1917	27		55		82	
≥75	447	6		43		49	
Surgical resection			0.546		0.061		0.186
Total	1264	16		60		76	
Distal	1045	16		38		54	
Proximate	55	1		0		1	
Reconstruction			0.499		0.063		0.203
Roux-en-Y	1264	16		60		76	
B-I	879	13		32		45	
B-II	166	3		6		9	
Esophagogastric	55	1		0		1	
Lymphadenectomy			0.567		0.485		0.367
D1/D1+	450	5		16		21	
D2	1914	28		82		110	
No. of resected LNs		0.861		0.292		0.407	
<33	1325	18		60		79	
≥33	1039	15		38		53	

*BMI* body mass index, *IBL* intraoperative blood loss

### Incidence and characteristics of SSIs

**Fig. 1:**
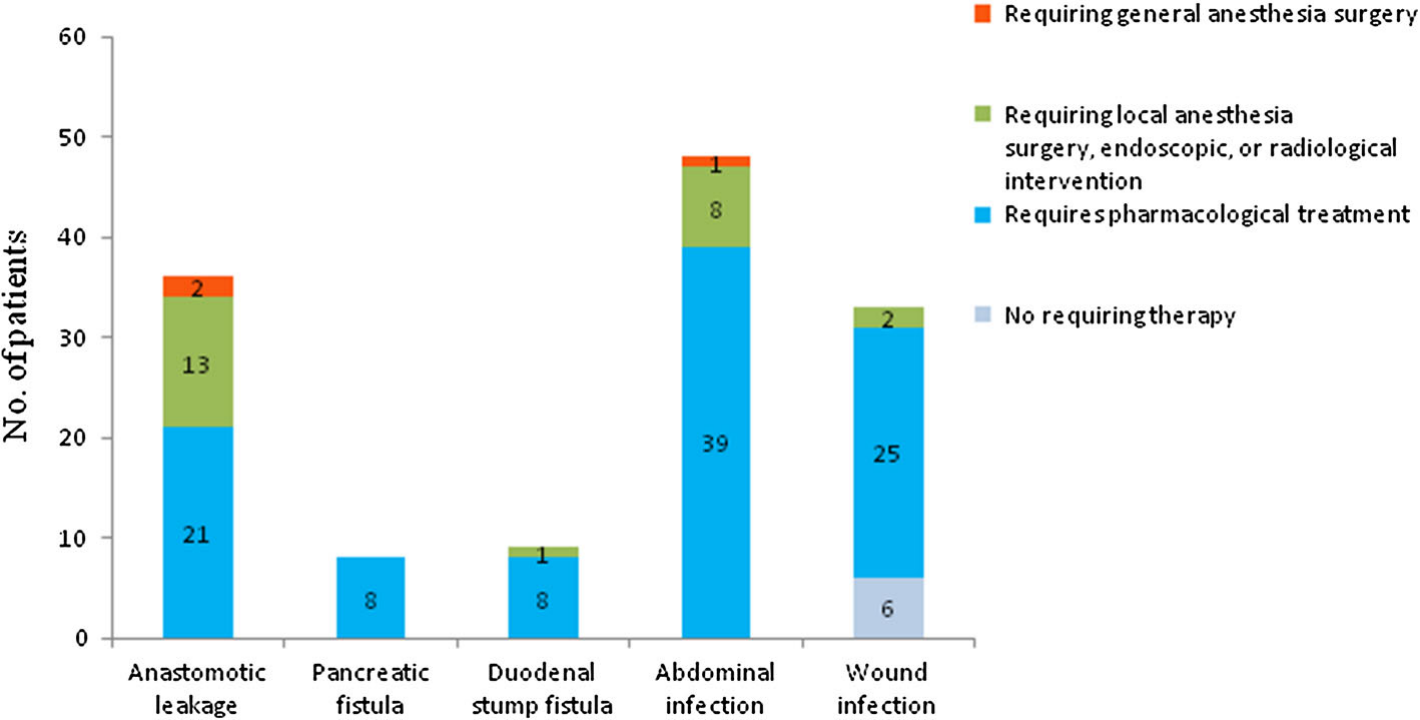
Rates of the SSIs and the treatments for the SSIs

Of 2364 patients, intraoperative complications were observed in 25 patients (1.1 %). Postoperative complications were observed in 330 patients (14.0 %) (Table 2), among which SSIs (all incisional and organ/space SSIs were grouped together) were present in 131 patients. A total of 33 (1.4 %) patients had incisional SSIs, including 29 superficial incisional SSIs and four deep incisional SSIs. A total of 98 (4.1 %) patients had organ/space SSIs. Thirty-three of the 98 organ/space SSIs were intra-abdominal abscesses due to anastomotic leakage; nine resulted from duodenal stump fistula, five resulted from pancreatic fistula, three were abscesses resulting from both pancreatic fistula and anastomotic leakage, and the cause of organ/space SSIs was unknown in 48 patients. Seventy-one of the 98 organ/space SSIs required anti-infection treatment, 24 required endoscopic or radiological intervention, and three required general anesthesia during surgery (two anastomotic leakages and one intra-abdominal abscess). Six of the 33 incisional SSIs only required dressing changes, 25 required anti-infection treatment, and two required resuturing (Fig. 1). The mean lengths of the postoperative hospital stay of patients with non-SSI were 12.30 ± 5.18 days, and of patients with overall SSIs, superficial incisional SSIs, and organ/space SSIs were 27.69 ± 16.56, 18.27 ± 8.80, and 30.87 ± 17.37 days, respectively. Four patients (0.2 %) died by the 30th postoperative day. The following causes of death were noted: intra-abdominal abscesses due to anastomotic leakage (two patients); pancreatic fistula and anastomotic leakage (one patient); and organ/space SSIs with unknown cause (one patient). And by the 90th postoperative day, the deaths added up to eight patients (0.3 %). Complications associated with SSIs were anastomotic bleeding, abdominal bleeding, chylous leak, sepsis, pneumonia, and transient liver enzyme abnormalities (Table 2).

**Table 2:** Intraoperative and postoperative morbidity associated with surgical site infections

	No. Patients *N* (%)	With SSIs *N* (%)	OR	*p*
Intraoperative morbidity	25 (1.1)	3 (0.1)	2.355 (0.696–7.973)	0.168
Vascular injury	13 (0.6)	2 (0.1)	3.132 (0.687–14.277)	0.140
Spleen injury	7 (0.3)	1 (0.0)	2.855 (0.341–23.891)	0.333
Transverse colon injury	3 (0.1)	0	0	0.999
Jejunum injury	2 (0.1)	0	0	0.999
Postoperative morbidity	330 (14.0)	/	/	/
Incisional SSIs	31 (1.3)	/	/	/
Organ/space SSIs	98 (4.1)	/	/	/
Anastomotic bleeding	11 (0.5)	3 (0.1)	6.519 (1.709–24.865)	0.006
Abdominal bleeding	18 (0.8)	7 (0.3))	11.403 (4.346–29.923)	0.000
Ileus	24 (1.0)	2 (0.1)	1.558 (0.362–6.698)	0.551
Anastomotic stricture	3 (0.1)	0	0	0.999
Remnant gastric stasis	25 (1.1)	0	0	0.998
Chylous leak	21 (0.9)	7 (0.3)	8.948 (3.548–22.568)	0.000
Sepsis	5 (0.2)	4 (0.2)	70.299 (7.800–633.557)	0.000
Adhesive intestinal obstruction	1 (0.0)	0	0	0.999
Infarct of spleen	1 (0.0)	0	0	0.999
Pneumonia	137 (5.8)	35 (1.5)	7.617 (4.930–11.768)	0.000
Arrhythmia	6 (0.3)	0	0	0.999
Cardiac failure	3 (0.1)	0	0	0.999
Transient liver enzyme abnormalities	8 (0.3)	2 (0.1)	5.755 (1.150–28.792)	0.033
Urinary tract infection	11 (0.5)	2 (0.1)	3.447 (0.747–15.893)	0.113
Catheter-related infection	4 (0.2)	0	0	0.999
DIC	4 (0.2)	0	0	0.999
Cerebral infarction	1 (0.0)	0	0	0.999

*SSIs* surgical site infections; *DIC* disseminated intravascular coagulation

### Univariate and multivariate analyses associated with the SSIs

Tables 1 and 3 show the results of the univariate and multivariate analyses of the possible risk factors for the development of SSIs. No statistically significant factors were associated with incisional SSIs in the univariate analyses. In addition, five factors were associated with an increased risk of organ/space SSIs, including the BMI (*p* < 0.001), Charlson co-morbidity score (*p* = 0.002), perioperative transfusion (*p* < 0.001), operation time ≥240 min (*p* < 0.001), and intraoperative blood loss (*p* < 0.001). We evaluated the risk factors for organ/space SSIs by multivariate analysis. The multivariate analysis revealed that the following were adverse risk factors for organ/space SSIs: BMI ≥ 25 kg/m^2^ (OR = 3.638, *p* < 0.001), intraoperative blood loss ≥ 75 ml (OR = 2.071, *p* = 0.010), operation time ≥ 240 min (OR = 3.865, *p* < 0.001), and perioperative transfusion (OR = 3.131, *p* < 0.001).

**Table 3:** Multivariate analysis associated with organ/space SSIs

Variables	β	OR	95 % CI	*p*
BMI ≥ 25 kg/m^2^	1.291	3.638	2.135–6.199	<0.001
Operative time ≥ 240 min	1.352	3.865	2.137–6.990	<0.001
IBL ≥ 75 ml	0.728	2.071	1.192–3.597	0.010
Perioperative transfusion	1.141	3.131	1.798–5.450	<0.001

*β* regression coefficients, *BMI* body mass index, *IBL* intraoperative blood loss

### The scoring system for organ/space SSIs

Despite the differences in the regression coefficients, which ranged from 0.728 to 1.352 for organ/space SSIs, respectively, 1 point was assigned for each of the risk factors for simplicity. The resulting BBOT (BMI, blood loss, operation time, and transfusion) scores were built for organ/space SSIs. Because only five of the patients had 4 points, the following four risk groups were established: low risk (0 points, i.e., no risk factors), intermediate risk (1 point, i.e., one risk factor), high risk (2 points, i.e., two risk factors), and extremely high risk (3 or 4 points, i.e., three or four risk factors). The distribution of the patients according to the scoring system was as follows: low risk, 59.0 %, intermediate risk, 28.2 %, high risk, 10.3 %, and extremely high risk, 2.5 %. The incidence rates of organ/space SSIs among the patients in the low-, intermediate-, high-, and extremely high-risk categories were 1.8, 3.9, 9.9, and 39.0 %, respectively (*p* < 0.001).The relative risks of organ/space SSIs in the intermediate-, high-, and extremely high-risk groups compared with the low-risk group were 2.136 (95 %CI, 1.101–4.145, *p* = 0.025), 5.869 (95 % CI, 2.960–11.635, *p* < 0.001), and 34.027 (95 % CI, 15.570–74.360, *p* < 0.001), respectively (Table 4).

**Table 4:** BBOT scoring system for organ/space SSIs

Risk group	BBOT score	No. patients (*n* = 1653 %)	No. patients (*n* %)	OR	95 % CI	*p*
Low	0	975 (59.0)	18 (1.8)	1	/	/
Intermediate	1	466 (28.2)	18 (3.9)	2.136	1.101–4.145	0.025
High	2	171 (10.3)	17 (9.9)	5.869	2.960–11.635	<0.001
Extremely high	≥3	41 (2.5)	16 (39.0)	34.027	15.570–74.360	<0.001

BBOT, BMI, blood loss, operation time, and transfusion

### Discrimination

**Fig. 2:**
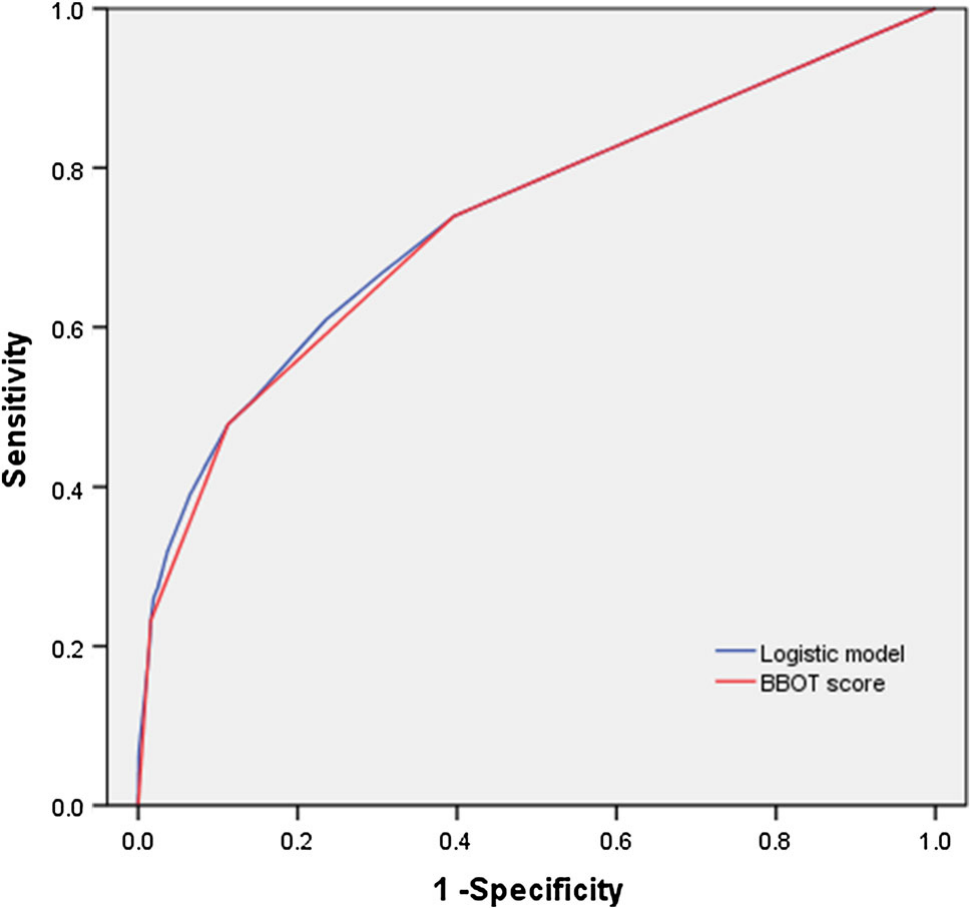
Receiver operating characteristic curves for prediction of organ/space SSIs after laparoscopic gastrectomy in the development sets

The final models discriminated the development sets with the areas under the receiver operating characteristic (ROC) curve. The area under the ROC curve was 0.739 (0.669–0.808) for the logistic regression model and 0.734 (0.665–0.803) for the simplified BBOT score for organ/space SSIs (Fig. 2). To evaluate the models’ performance, the observed versus predicted incidence rates in the validation set were compared. The predicted incidence rates for the low-, intermediate-, high-, and extremely high-risk categories in the validation set were 1.9, 3.9, 10.0, and 39.0 %, respectively. There were no statistically significant differences found between the observed and BBOT predicted incidence rates for organ/space SSIs in the validation set (*p* > 0.05) (Table 5).

**Table 5:** Observed and BBOT predicted incidence rates for organ/space SSIs in the validation set

Risk group	Observed organ/space SSIs (%)	BBOT predicted organ/space SSIs (%)	*p*
Low	2.3	1.9	0.590
Intermediate	3.0	3.9	0.587
High	8.8	10.0	0.772
Extremely high	38.9	39.0	>0.999

## Discussion

SSIs are one of the most common nosocomial infections, and they are a fundamentally important clinical outcome indicator in elective surgery [12–15]. Effectively decreasing the incidence of SSIs is a global challenge. In 2002, the Surgical Infection Prevention project (SIP) was initiated under the direction of the CMS and CDC [16]. The aim of SIP was to reduce the nationwide incidence of SSI through systems level protocol implementation. The SIP evolved into the Surgical Care Improvement Project (SCIP), which was a joint effort between the CMS and Joint Commission to further improve the nationwide compliance with standards of care in surgical practice. Some sites have demonstrated decreased incidence of SSIs associated with improved compliance in SCIP measures [17, 18]. However, the incidence of SSIs has failed to decrease substantially over time on a national scale [19, 20]. Therefore, it is particularly important to identify and prevent the risk factors for SSIs. The incidence rates of overall SSIs, incisional SSIs, and organ/space SSIs after tradition open gastrectomy are 7.0–20.8, 1.7–8.6, and 5.1–13.3 %, respectively [3–6]. To the best of my knowledge, no reports have been designed to identify the risk factors for SSIs after a laparoscopic radical gastrectomy for gastric cancer. In the present study, laparoscopic gastrectomy was initially performed in patients diagnosed with cT1N0M0–cT2N0M0 gastric cancer. With the experience accumulation and expanded use of laparoscopic gastrectomy, the indications were then gradually extended to more advanced stages of disease. And the incidence rates of overall SSIs, incisional SSIs, and organ/space SSIs after traditional open gastrectomy were 5.5, 1.4, and 4.1 %, respectively. Moreover, 4.1 % (4/98) of patients with organ/space SSIs died by the 30th postoperative day. As a result, investigating the risk factors for organ/space SSIs and selecting risk-adapted interventions may help reduce the incidence rates of organ/space SSIs.

Previous studies have reported several risk factors for SSIs after open gastrectomy, such as advanced age, a BMI of 25 or higher, diabetes mellitus, a longer operation duration, blood loss, total gastrectomy, and combined resection procedures [4, 6, 21]. However, laparoscopic gastrectomy has its own characteristics, and the aforementioned risk factors have provided limited reference value for this procedure. And we found that the perioperative transfusion, operation time ≥240 min, intraoperative blood loss ≥75 ml, and BMI ≥ 25 kg/m^2^ were the risk factors associated with the incidence of organ/space SSIs after laparoscopic gastrectomy. Intraoperative blood loss requires additional hemostasis by ligation and compression, and a massive hemorrhage might lead to hypovolemia; these conditions appear to be associated with poor wound healing and increased infection rates from hypoxia [22–24]. Furthermore, the cases of preoperative anemia, intraoperative or postoperative blood loss that require allogeneic blood transfusion typically induce immunosuppression and predispose patients to postoperative infection. Allogeneic leukocytes may play a critical role in the induction of transfusion-induced immunosuppression [25–28]. The operation time depends on various parameters, such as the surgeon’s experience and technical or intraoperative problems (e.g., accidental puncture of an intra-abdominal organ, intraoperative hematoma, or organ lesions). Increasing the length of the procedure theoretically increases the susceptibility of the wound by increasing bacterial exposure and the extent of tissue trauma (more extensive surgical procedure) and decreasing the tissue level of the antibiotic [29, 30]. In addition, there is more surrounding tissue to separate and dissect in patients who have a high BMI. These patients typically have significantly higher rates of SSIs as well as conversion to open surgery and postoperative complications [6].

Few studies have been designed to create a scoring system for predicting the risk of SSIs after an open procedure. Among these systems, the National Nosocomial Infections Surveillance (NNIS) basic risk index is one of the most widely used systems to predict the risk of SSIs. The NNIS basic SSI risk index consists of the following three criteria: American Society of Anesthesiologists score of 3, 4, or 5; wound class; and duration of surgery. Moreover, it has proven to be useful for risk adjustment for many open procedures. However, Gaynes [7] also noted that the use of a laparoscope is accompanied by significantly lower rates of SSIs after gastric surgery, and the NNIS basic SSI risk index might be not suitable for laparoscopic gastrectomy. Our BBOT scoring system was based on the final logistic regression model. With respect to the risk stratification for organ/space SSIs, the BBOT scoring system classified the patients after laparoscopic gastrectomy into four groups and identified the extremely high-risk group, which had a 23.8-fold higher risk of organ/space SSIs than that of the lowest risk group. The BBOT scoring system discriminated the development sets with an area under the ROC curve of 0.734, which is similar to the logistic regression model. There were no statistically significant differences between the observed and BBOT scoring system predicted incidence rates in the validation set, indicating that this system performed well. Patient and disease characteristic data are routinely available, which might have implications for selecting risk-adapted interventions to improve surgical safety. Since only the BMI can be identified preoperatively, overweight patients (BMI ≥ 25 kg/m^2^) might be referred to operators with more experience to cut operation time and reduce blood loss. Also, if one or more of their other risk factors occurs intraoperatively or postoperatively, such as perioperative transfusion, operation time ≥240 min, intraoperative blood loss ≥75 ml, it is necessary to be aware of the sign and symptom closely and must be examined by laboratory tests and imageological examinations postoperatively, in order to early detection and treatment of SSI.

Our study has some limitations. Firstly, as with other retrospective studies, inherent selective bias is inevitable, although we use a prospectively collected database. Second, there are only 14.3 % patients with BMI ≥ 25 kg/m^2^ in our study, whereas one-third of the US population had a BMI of 27 kg/m^2^ or greater [31]. So it would seem that this scoring system should be validated by Western centers before applying it in Westerners.

In conclusion, our BBOT scoring system allows for easy and validated risk stratification of the organ/space SSIs in the clinical setting. This stratification might be helpful for selecting risk-adapted interventions that reduce the rates of organ/space SSIs and improve the surgical safety. A prospective multiple-center study with a large series would help validate this scoring system.
